# Organic Food Purchases in an Emerging Market: The Influence of Consumers’ Personal Factors and Green Marketing Practices of Food Stores

**DOI:** 10.3390/ijerph16061037

**Published:** 2019-03-22

**Authors:** Hoang Viet Nguyen, Ninh Nguyen, Bach Khoa Nguyen, Antonio Lobo, Phuong Anh Vu

**Affiliations:** 1 Department of Research Administration, Thuongmai University, Hanoi 100000, Vietnam; nhviet@tmu.edu.vn; 2 Business Sustainability Research Group, Thuongmai University, Hanoi 100000, Vietnam; 3 Department of Entrepreneurship, Innovation and Marketing, La Trobe Business School, La Trobe University, Melbourne, VIC 3086, Australia; 4 Faculty of Marketing, Thuongmai University, Hanoi 100000, Vietnam; nbkhoa@tmu.edu.vn (B.K.N.); vphuonganh@gmail.com (P.A.V.); 5 Department of Management and Marketing, Swinburne Business School, Swinburne University of Technology, Melbourne, VIC 3122, Australia; alobo@swin.edu.au

**Keywords:** organic food, green marketing, food stores, personal factors, contextual factors, environmental influences, price barriers, emerging market, Vietnam

## Abstract

The consumption of food has a significant impact on the environment, individuals and public health. This study aims to investigate the integrative effects of consumers’ personal and situational factors on their attitude and purchase behavior of organic meat. The consumption of this product has been widely regarded as contributing towards sustainable food practices. The study was conducted in an emerging market economy, i.e., Vietnam. Data were collected using a customized and validated survey instrument from a sample of 609 organic meat consumers at four food outlets in Hanoi. The findings suggested that consumers’ concerns regarding the environment, health, food safety and their knowledge of organic food, all significantly impacted their attitude towards the purchase behavior of organic meat. Interestingly, their positive attitude did not necessarily translate into their actual purchase of organic meat. Additionally, food stores’ green marketing practices significantly enhanced consumers’ actual purchase behavior. Conversely, premium prices of organic meat were certainly a deterrent for the actual purchase of organic meat. The findings of this study have several important implications for organic food producers, retailers, policy makers and socio-environmental organizations that seek to develop intervention strategies aimed at increasing organic meat consumption in Vietnam.

## 1. Introduction

It is well known that the consumption of food has a significant impact on the environment, individuals and public health [1,2,3]. Notably, food consumption is associated with environmental issues such as increased greenhouse gas emissions, water scarcity and pollution [2]. For example, the consumption of beef has a substantial impact on the ecosystem. This is owing to the fact that the production of protein generates a relatively high amount of carbon dioxide, e.g., an equivalent of 221.63 g of carbon dioxide is generated per gram of protein [4,5]. Consuming food containing undesirable residues and microorganisms causes severe individual health problems, e.g., pain, illness and death [6,7]. In addition, food-borne diseases result in medical costs and losses to the public health sector [8]. Hence, promoting and accelerating the adoption of more sustainable food behaviors is of the utmost importance for enhancing environmental sustainability as well as individual and public well-being. Sustainable food behaviors include activities such as purchasing and consuming organic food, eating less unhealthy food, eating local food and preparing food that has less wastage [9,10]. Importantly, the promotion of these behaviors should be prioritized in developing and emerging countries which are facing serious environmental problems and a colossal increase in food consumption [9,11]. The growth in population and income has driven consumer demand for food products, and this is especially true in developing and emerging countries, particularly for healthy and environmentally friendly food [12].

Organic food purchase and consumption has been widely regarded as contributing towards sustainable behavior [13,14]. This is partly driven by consumers’ socio-environmental responsibility in addition to their personal interest and choice [10]. Although there exist various definitions of organic food, it can be broadly defined as products which are “grown without the use of pesticides, synthetic fertilizers, sewage sludge, genetically modified organisms, or ionizing radiation” as well as products produced “free of antibiotics or growth hormones” [15] (p. 195). The majority of consumers believe that organic food is eco-friendly, healthier, safer, cleaner, more nutritious, tastier and safer as compared to conventional food [13,16,17,18].

A considerable number of studies on organic food have focused on consumers’ personal factors that motivate attitude and purchase behavior associated with organic food [19]. Key personal factors include values, environmental concern, knowledge, perceived quality, emotions, health consciousness, concerns with respect to nutrition, food taste and food safety [20,21,22]. Interestingly, there exists mixed findings regarding the relationship between consumers’ attitudes and their purchase of organic food [23]. While various studies demonstrate that consumers’ attitudes towards organic food significantly enhance their purchase intention and behavior, several authors report that many consumers do not actually buy organic food despite displaying positive attitudes towards them [24,25]. Aschemann-Witzel and Niebuhr Aagaard [23] note that young consumers hold highly favorable attitudes about organic food, but their actual purchases remain low. According to Padel and Foster [26], such an anomaly can be explained by the complexity of the consumer decision-making process and the varied motives and barriers associated with different types of organic food. This finding is extended by Vermeir and Verbeke [10] who suggest that marketing factors such as price, product quality, convenient distribution and brand familiarity remain the most important criteria in the consumer decision-making process. These factors along with consumer habits of buying conventional produce may dilute the impact of attitudes toward organic food on actual purchase behavior. Hence, although consumers might believe that organic food offers environmental and health benefits and that the purchase of organic food is beneficial, they may be unable to buy, or they may decide not to buy the product owing to its high price, lack of availability, poor labelling and mediocre point-of-purchase display. Several authors assert that organic food retailers need to develop and implement effective green marketing practices to support consumers’ decision-making process [17,27]. Green marketing practices may include the production and distribution of environmentally friendly products (e.g., organic food), marketing communications for green products (e.g., advertising, sales promotion, public relations and publicity), eco-labelling and branding [17,28]. Nevertheless, these situational and environmental factors, unlike personal determinants, have been largely unexplored.

This study seeks to contribute to the literature on environmentally sustainable behavior and organic food consumption by investigating the integrative effects of consumers’ personal factors and their situational context on their attitude and purchase behavior associated with organic food. The situational context comprises of both motivators and hindrances in the development of organic food behavior [23]. The situational factors also represent the external environment’s impact on the opportunity to perform the behavior [29]. Hence, particular emphasis is placed on how food stores’ green marketing practices and price barriers enhance or impede a consumer’s purchase of organic food products. Green marketing practices have been emphasized because they include various activities associated with distributing, communicating and promoting eco-friendly products. Krystallis and Chryssohoidis [30] assert that price is the most important criterion considered by consumers when purchasing food. The high price of organic food has been identified as the most relevant barrier to organic food purchase and consumption in previous studies [16,22]

This study also aims to enrich the literature on organic food behavior in developing and emerging markets, which have received less attention from scholars [11]. By focusing on an emerging market, the study examines whether or not the attitude-behavior gap previously identified in developed countries is relevant in the new research context, i.e., Vietnam. This Southeast Asian country is a typical emerging market with a population exceeding 95 million and an annual average gross domestic product growth of 6% in the past decade [31]. Data from the Vietnam Household Living Standards Survey demonstrates that Vietnamese people spend approximately half of their income on food and beverage products [32]. Promoting sustainable food consumption has been among the country’s top priorities, aimed at addressing growing concerns about its environmental problems, health and food safety issues [9,33]. Many Vietnamese consumers associate sustainability with healthy food [9]. They also believe that unsafe food practices put the health of the public at risk [34]. Despite their concerns about the high price and poor labelling of safe and healthy foods like organic products, a number of Vietnamese consumers are willing to purchase and consume more organic food [9]. The government has implemented several initiatives such as the Decree 109/2018/ND-CP on organic culture and food safety standards to foster the development of the organic food industry. Manufacturers and retailers have put great efforts in offering diversified organic food products (e.g., vegetables, grains and meat) as well as expanding their distribution network [35,36].

The remainder of this paper is organized as follows. A detailed literature review comprising of the theoretical background and hypotheses development are initially presented. Thereafter, the research methodology is described, followed by the data analysis and reporting of key findings. A comprehensive discussion of the findings and their implications is then provided. Finally, conclusions, limitations and future research directions are presented.

## 2. Literature Review and Research Hypotheses

### 2.1. Theoretical Background

In general, consumers who hold favorable attitudes towards the purchasing of green products including organic food tend to make actual purchases [37]. The widely-accepted association between attitude and behavior has been explained by several theories such as the knowledge-attitude-behavior theory, the Alphabet theory and extended models of the Theory of Planned Behavior (TPB). The knowledge-attitude-behavior model postulates that environmental-related knowledge leads to environmental attitudes, and that this in turn motivates pro-environmental behavior [38]. Several researchers extending the TPB suggest that attitude affects actual behavior directly and indirectly via behavioral intention [37,39,40,41]. Zepeda and Li [42] develop the Alphabet theory by explaining consumers’ motivations for organic food consumption by integrating key elements of the value-belief-norm (VBN) model and the attitude-behavior-context (ABC) theory with knowledge and habit. Importantly, this theory emphasizes the role of context and habit in explaining the attitude-behavior relationship.

**Figure 1:**
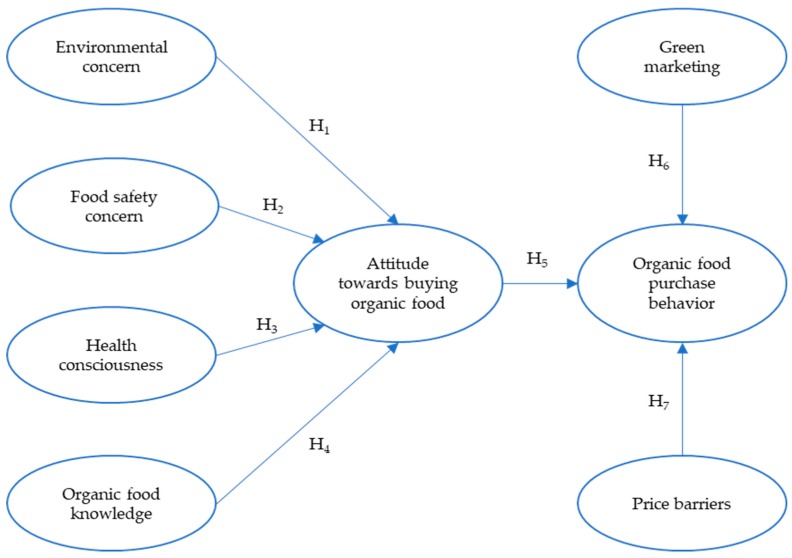
The proposed research model.

Given the important role of attitudes in enacting behavior, several authors stress the need for a better understanding of antecedents to organic food attitudes [21,43]. On the other hand, another research strand questioning the importance of attitudes, emphasizes the necessity to explain why consumers’ attitudes are not translated into their actual purchase of organic products [23]. In an effort to address the attitude-behavior gap and extend prior research on the antecedents to organic attitude, this study develops a unique model explaining organic food purchase as illustrated in Figure 1. This model combines the antecedent-attitude-behavior hierarchy with situational context factors including food stores’ green marketing practices and price barriers. The three antecedents of environmental concern, food safety concern and health consciousness have been identified as the most important determinants of organic food attitudes [21]. Organic food knowledge is also examined to provide further insight into the knowledge-attitude relationship, previously identified as a gap in the literature [44]. The hypothetical relationships between these constructs depicted in Figure 1 are discussed in the subsequent sections.

### 2.2. Development of Hypotheses

#### 2.2.1. Environmental Concern

According to Dunlap and Jones [45], environmental concern denotes “the degree to which people are aware of problems regarding the environment and support efforts to solve them or indicate the willingness to contribute personally to their solution” (p. 482). In general, consumers who are concerned about the environment tend to develop positive environmental attitudes, express willingness to pay more for eco-friendly products and exhibit pro-environmental behavior [46,47].

It is widely acknowledged that the production and consumption of food products, especially meat such as beef and pork, contribute to air pollution, land and water scarcity, and domestic waste to the ecosystem, leading to environmental degradation [48]. Environmental concern therefore appears to be a driving factor of organic food purchase behavior, and this has been largely attributed to being environmentally friendly [17]. Squires et al. [49] suggest that organic food buyers express interest in protecting the ecology and natural production process. Empirical studies conclude that environmental concern exerts a positive influence on attitude towards organic food purchases in both developed and developing countries such as Australia [13], Taiwan [50] and India [11]. Based on the above discussion, the following hypothesis has been developed:

**Hypothesis** **1.** *Consumers’ environmental concern has a positive impact on their attitudes towards buying organic food.*

#### 2.2.2. Food Safety Concern

Given the continuous occurrences of food safety incidents and food-related diseases [7], food safety has been identified as the top concern among consumers [51]. Food safety concern, in its broadest sense, indicates the degree to which people are worried about pesticide residues contained in food as well as about food scares [52] (p. 4). Essentially, consumers often associate food safety issues with the use of pesticides, fertilizers, antibiotics, artificial additives and preservatives in the food production process [3,43]. Organic production methods are considered as being free of these undesirable chemicals [21]. Van Loo et al. [53] point out that habitual buyers of organic chicken strongly believe that such a product has fewer residues. Michaelidou and Hassan [43] assert that food safety concern is the most relevant factor explaining consumer attitude towards organic food. Based on the aforementioned discussion, the following has been hypothesized

**Hypothesis** **2.** *Consumers’ food safety concern has a positive impact on their attitudes towards buying organic food.*

#### 2.2.3. Health Consciousness

Health consciousness reflects individuals’ thoughts on health issues and their readiness to undertake actions to ensure their health [50,52]. Consumers have been increasingly concerned about health and nutrition in food [1]. There is a general belief that organic foods are healthy to eat because such products are rich in nutrition and are chemically free [54]. Bryła [16] asserts that Polish consumers perceive healthiness as being the most important characteristic of organic food. A consumer survey conducted by Tsakiridou et al. [51] demonstrates that 87.6% of respondents perceive organic products to be healthier than conventional alternatives. Health consciousness is therefore a key determinant of organic food consumption [21]. Although Tarkiainen and Sundqvist [55] surprisingly find that health consciousness is not relevant in predicting attitude towards purchasing organic food, the majority of prior studies confirm a significant and positive relationship between these variables [43,56]. Importantly, Yadav and Pathak [11] assert that health consciousness is the strongest predictor of attitude towards organic food. Hence the following has been hypothesized:

**Hypothesis** **3.** *Consumers’ health consciousness has a positive impact on their attitudes towards buying organic food.*

#### 2.2.4. Organic Food Knowledge

It is a common belief that consumers’ awareness and knowledge about organic food play an important role in their organic purchasing decisions [57]. Several researchers regard the lack of knowledge concerning organic food as a barrier to organic food purchase [58]. Organic food knowledge entails what consumers know about organic food and their ability to judge the quality and unique characteristics of organic food products. Two major types of organic food knowledge include subjective and objective knowledge. While the former refers to what people perceive that they know, the latter denotes what people actually know about organic food [44]. Aertsens et al. [44] find that both objective and subject knowledge are positively related to attitudes towards organic vegetable consumption. These authors also suggest a causal relationship in that objective knowledge enhances subjective knowledge, which in turn improves one’s attitude towards organic purchase and consumption. Likewise, de Magistris et al. [57] confirm a positive association between consumers’ self-reported organic knowledge and their attitudes towards organic produce. Hence the following hypothesis has been formulated:

**Hypothesis** **4.** *Consumers’ organic food knowledge has a positive impact on their attitudes towards buying organic food.*

#### 2.2.5. Attitudes Towards Buying Organic Food

Attitudes associated with organic food and organic purchase have been central to research on organic food purchase and consumption [18,51]. Consumers’ attitudes towards buying organic food denotes their favorable or unfavorable evaluation towards purchasing organic food. Consumers who hold positive attitudes towards organic food believe that purchasing organic food is important and is a good choice [18]. Using the probit model analysis, Aertsens et al. [44] find a significant positive relationship between consumers’ attitudes about organic food consumption and the proportion of organic food consumed by them. Similarly, a regression analysis conducted by Dahm et al. [15] indicates that students who hold positive attitudes toward organic food actually consume more organic food at home, on campus and at restaurants. Additionally, a structural model developed by von Meyer-Hofer et al. [37] demonstrates a significantly direct relationship between attitude towards purchasing organic food and purchase behavior among German consumers. Hence, the following has been hypothesized:

**Hypothesis** **5.** *Consumers’ attitudes towards purchasing organic food has a positive impact on their purchase behavior.*

#### 2.2.6. Green Marketing Practices

Marketing with an environmental perspective has been referred to as ‘green marketing’ ‘environmental marketing’, ‘socially responsible marketing’ and ‘sustainable marketing’ [59,60]. In general, green marketing includes “marketing activities which attempt to reduce the negative social and environmental impacts of existing products and production systems, and which promote less damaging products and services” [61] (p. 129). In this present study, food stores’ green marketing practices refer to eco-labelling, providing an environmentally-friendly shopping environment, advertising and promoting organic food using in-store promotional tools including fliers as well as selling various brands of organic food.

It has been widely acknowledged that green marketing practices significantly affect consumers’ choice of eco-friendly products [62]. A Malaysian study conducted by Mohd Suki [27] reveals that stores’ green marketing practices enhance consumer perception about organic food quality and image. Verhoef [22] finds that marketing variables such as product quality and distribution positively affect consumer purchase of organic food. Hence, the following hypothesis has been formulated:

**Hypothesis** **6.** *Food stores’ green marketing practices have a positive impact on consumer attitudes towards buying organic food.*

#### 2.2.7. Price Barriers

Price (monetary) barriers constitute the critical hindrance to increasing consumer demand for organic food [16,22,63]. Price barriers refer to consumers’ perception of organic food price and their ability and willingness to buy such a product despite the high price [14]. In a consumer survey carried out by Xie et al. [64], about 82% of the respondents indicate that high price premium is the reason for not buying organic products. In general, the majority of consumers are not willing to pay a price premium above 10–20% for organic food [65,66]. Van Doorn and Verhoef [67] argue that the high price of organic food negatively affects consumer-perceived value (i.e., cost versus benefits) of such a product. Tanner and Kast [14] find a strong and negative correlation between price barriers and consumer purchase of different organic food products. Hence the following has been hypothesized:

**Hypothesis** **7.** *Price barriers have a negative impact on consumer attitudes towards buying organic food.*

## 3. Research Methodology

### 3.1. Researched Product Category

Determinants influencing organic food attitudes and behavior may vary across different categories of organic food [31]. Organic meat was intentionally chosen as the researched food product category owing to several reasons. Firstly, while a considerable number of studies have investigated specific categories of organic food such as organic fruits and vegetables, the organic meat category has been largely unexplored. Secondly, given that Vietnamese people’s expenditure on meat constitutes 14% of their total food expenditure [32], meat is among the most popular food products consumed in the country. Given that meat consumption is strongly linked to income [5], it is expected that Vietnamese consumers will consume more meat in the coming years. This is a particularly controversial issue since meat production and consumption is among the largest contributors to environmental problems, such carbon dioxide emissions and excessive land use [4]. Thirdly, antibiotic and veterinary residues in meat have received widespread attention from Vietnamese consumers and media [34]. This may be a driving factor for organic meat consumption.

### 3.2. Measures

The items operationalizing the constructs in the research model were selected and adapted from measurement scales validated in prior research. The original scales in English were translated into Vietnamese using the back-translation suggested by Usunier [68]. Accordingly, a professional translator performed the translation of the survey instrument from English to Vietnamese, and this Vietnamese version was then translated back into English by another translator, who worked independently without being informed of the first translation. As there were minor discrepancies between the original survey and the back-translated survey, 2 bilingual marketing professors were invited to review the translations. These professors also worked with the translators to discuss and reconcile the discrepancies until an agreement was reached on the most suitable version of the survey instrument, so as to ensure semantic equivalence. As most of the scales were developed for Western developed markets, 2 focus groups were conducted to identify the potential problems of using these items in the context of Vietnam. Participants in the first focus group included 6 organic meat consumers, whilst the second focus group consisted of 5 marketing professors. These focus groups were facilitated and moderated by the researchers. Following the focus groups, some items were revised and modified. For example, the participants suggested to clarify the second item measuring food safety concern by adding antibiotics and veterinary to the original item. In addition, a new item was suggested for operationalizing organic food knowledge. All the items, except for purchase behavior, were measured using a 7-point Likert scale, which ranged from 1 for ‘Strongly disagree’ to 7 for ‘Strongly agree’.

For *environmental concern*, 4 items were adopted from Yadav and Pathak’s [11] study. To measure *food safety concern,* 3 items were taken from Michaelidou and Hassan [43]. Another 3 items operationalizing *health consciousness* were adapted from Tarkiainen and Sundqvist [55]. A total of 4 items were used to measure *organic food knowledge*. Of these, 3 items were adopted from Aertsens et al. [44] and 1 item regarding the environmental and health benefits of organic meat was suggested by the focus groups. *Attitude towards buying organic food* was operationalized using 4 items from prior research conducted by Arvola et al. [69] and Dean et al. [70]. Another 4 items operationalizing *green marketing practices* implemented by food stores were adopted from Mohd Suki [27]. *Price barriers* were operationalized using 3 items from studies conducted by Tanner and Kast [14] and Verhoef [22].

To measure the *purchase behavior* of organic food, 1 item seeking respondents’ purchase frequency was adopted from Dean et al. [70] (‘How often in the last 10 times you purchased meats were [they] organic ones?’). There were 7 response categories, anchored at 1 for ‘Never’ and 7 for ‘Always’.

### 3.3. Ethics Considerations

The importance of adhering to ethical standards with respect to human research activity has been addressed in this study. The research ethics application, which is a part of the research project application, was submitted to the Vietnam National Foundation for Science and Technology Development (NAFOSTED) in March 2018. The application describes details of the research project, including participant recruitment, data collection procedures, description of anticipated risks, data storage and security, and informed consent. The project (NAFOSTED 502.02-2018.303) was reviewed and approved on 5 September 2018.

The research project followed the approved protocol. Respondents notified the research purpose and their informed consent by reading the cover page of the survey and by listening to the interviewers. Participation was on a voluntary basis, in which respondents could withdraw at any time. Completion of the survey was taken as a respondent’s ‘informed consent’ to participate in the research. The process ensured the respondent’s anonymity, confidentially and privacy. Only anonymous and aggregated data were captured and subsequently reported in the research report and in published conference papers and journal articles.

### 3.4. Data Collection and Sample

The respondents in this study were Vietnamese citizens aged 18 and over, who had purchased organic meat. This enables the investigation of consumers’ actual purchases of organic meat. Paper-based surveys were used to collect data from eligible consumers at 4 food stores in the capital city of Hanoi, which is located in the north of Vietnam. The paper-based approach has proven effective in several research projects on pro-environmental behavior and organic food in Vietnam [33,41]. Consumers in major cities such as Hanoi have been the subject of prior relevant studies because they have higher income and tend to pursue a sustainable lifestyle [9,47,71,72]. Furthermore, supermarkets and food stores are the main distributors of organic food products including organic meat in big cities like Hanoi and Ho Chi Minh [36]. Experienced and trained research assistants acted as interviewers who were stationed at the stores to approach consumers after they had completed their purchases. Respondents were then initially asked the screening questions (i.e., age and past organic meat purchase) before being presented with the informed consent statement and were then requested to voluntarily provide their responses. A US$ 2.5 (Vietnamese Dong 50,000) cash incentive was offered to them in appreciation of their participation.

The data collection period lasted 3 months, during which time a total number of 635 surveys were returned. Data from the returned surveys were screened to examine potential missing data, outliers and normality of distribution. The standardized values (z scores) indicated that 4 surveys contained univariate outliers, while the Mahalanobis distance demonstrated that 22 surveys comprised multivariate outliers. The final effective sample therefore included 609 responses. Of these, 319 (52.4%) were female and 290 were males (47.6%). Additionally, the majority of the respondents (54.4%) were aged under 40 years, while 61.9% had higher education degrees. The demographic profile of the respondents is depicted in Table 1.

According to the latest data published by the General Statistic Office of Vietnam—GSO [73], the ratio of male to female was 49:51, which is quite similar to that of our sample. The Vietnam Intercensal Population and Housing Survey conducted by GSO [74] demonstrates that the percentage of persons aged 20–29, 30–39, 40–49, 50–59 and 60 and above was 18.9%, 15.8%, 13.4%, 10.3% and 10.1% respectively. Given that our study only focused on people aged 18 and above, it can be argued that our sample fairly resembles the percentages of the different age groups in the population. Furthermore, the marriage incidence of the respondents in our sample was 62%, which is slightly lower than that of people aged 18 and over in the population (i.e., 67%). Additionally, the educational levels and household monthly income of our sample are similar to those reported in earlier pro-environmental studies in Vietnam [41,75]. Notably, a majority of the respondents (38.9%) reported that their household incomes was VND 10,000,001–20,000,000 per month, which can be classified as being middle-high income earners [76]. Overall, it is reasonable to argue that our sample fairly represents the entire population of Vietnam.

## 4. Data Analysis

### 4.1. Statistical Approaches

Following the guidelines recommended by Hair et al. [77], confirmatory factor analysis (CFA) and reliability analysis using Cronbach’s alpha (α) were performed to assess construct validity and reliability. The CFA was also used to evaluate the validity of the measurement model. The hypotheses were then tested using structural equation modelling (SEM). Please note that as purchase behavior was measured by one item, this variable is not applicable to CFA. Two application software were used to perform the data analysis, i.e., SPSS and AMOS version 24 (IBM Corporation, New York, NY, USA).

The goodness-of-fit (GOF) of the measurement and structural models was examined using commonly-applied fit indices including χ^2^/*df* (chi-square to degree-of-freedom ratio), GFI (goodness-of-fit index), AGFI (adjusted goodness-of-fit index), CFI (comparative fit index), TLI (Tucker and Lewis index) and RMSEA (root mean square error of approximation). According to prominent studies such as those of Hu and Bentler [78] and Hair et al. [77], it is reasonable to conclude that the model fit is good when χ^2^/*df* < 3, the values of GFI, CFI, TLI ≥ 0.90 and RMSEA ≤ 0.08.

### 4.2. Measurement Model, Construct Reliability and Validity

To assess the measurement model fit, all constructs in the research model, except purchase behavior, were subjected to CFA using maximum likelihood estimation. The resultant statistics were all above the suggested level (χ^2^ (254) = 337.933, *p* < 0.001; χ^2^/*df* = 1.330; GFI = 0.958; AGFI = 0.946; CFI = 0.986; TLI = 0.983; RMSEA = 0.023). These GOF indices revealed a good model approximation to the sample data.

As recommended by Byrne [79] and Hair et al. [77], construct validity was examined using convergent and discriminant validity. Specifically, convergent validity was assessed based on three conditions, i.e., (1) standardized factor loadings values were above 0.5; (2) composite reliability (CR) was higher than the average variance extracted (AVE), and CR was above 0.7; and (3) AVE was above 0.5. As depicted in Table 1, the relevant data demonstrated strong convergent validity. Additionally, Table 2 shows that the square root of the AVE of each measure (0.732–0.766) was higher than its correlation coefficients with other constructs, indicating that discriminant validity is ensured [80]. Furthermore, all bivariate correlations between constructs were less than 0.5, hence possible problems of multi-collinearity were non-existent [81].

In addition, reliability analysis, as shown in Table 3, demonstrated that α values for constructs ranged from 0.777 to 0.848. Moreover, corrected item-to-total correlations were all greater than 0.5. It is therefore reasonable to conclude that all the measures have good internal consistency of reliability [82].

### 4.3. Hypotheses Testing

SEM was applied to test the seven proposed hypotheses. The resulting indices were χ^2^ (278) = 417.480, *p* < 0.001; χ^2^/*df* = 1.052; GFI = 0.949; AGFI = 0.936; CFI = 0.977; TLI = 0.974; RMSEA = 0.029. These GOF indices altogether revealed a good fitting model which explained the significant 31% of the variation in consumer purchase of organic meat.

The results of the hypotheses testing are illustrated in Table 4. As indicated, all the relationships between each of the variables were significant, except between attitude and purchase behavior. Hence, H1, H2, H3, H4, H6 and H7 were accepted, while H5 was rejected. Specifically, environmental concern (*β* = 0.273, *p* < 0.001), safety concern (*β* = 0.232, *p* < 0.001), health consciousness (*β* = 0.205, *p* < 0.001) and organic food knowledge (*β* = 0.097, *p* < 0.05) had a significant positive impact on attitude towards purchasing organic food. Interestingly, the relationship between attitude towards purchasing organic food and consumer purchase behavior was positive but non-significant (*β* = 0.049, *p* > 0.05). As expected, green marketing practices exerted a strong positive influence on consumer purchase of organic meat (*β* = 0.430, *p* < 0.001). In contrast, price barriers were negatively associated with such purchase (*β* = −0.194, *p* < 0.001).

## 5. Discussion, Implications and Limitations

This study has developed and validated a model combining key elements of several theories to explain consumer purchase of a specific category of organic food, i.e., meat. The SEM findings indicate that such an integrative model is useful in predicting consumer purchases of environmentally-friendly products (i.e., organic meat) in the context of emerging markets such as Vietnam. The importance of further research into organic food purchases in developing and emerging countries has been emphasized in the literature [21,52]. A notable finding in this study concerns the attitude-behavior gap in the context of organic food purchase and consumption. That is, Vietnamese consumers’ attitudes towards purchasing organic meat are not significantly translated into their actual purchase behavior. Whilst this finding diminishes the significance of attitudes in motivating organic food purchases, which has been found in numerous studies [15,37,44], it confirms the attitude-behavior gap in a new research context, i.e., organic meat purchase in Vietnam. This discrepancy could be attributed to the high price of organic food. Similar to earlier work [22], the findings reveal that price barriers negatively affect consumer purchase frequency of organic food. Specifically, consumers perceive that organic meat is too expensive and such a high price represents an obstacle to their purchase behavior. This can be explained by the widespread financial constraints of consumers in emerging markets [83].

Another key finding is that food stores’ green marketing practices significantly motivate consumer purchase behavior of organic meat. This extends the findings of prior studies in both developing and developed countries. While the Malaysian study conducted by Mohd Suki [27] demonstrates that green marketing practices enhance product image and corporate social responsibility, Dutch research carried out by Verhoef [22] finds that marketing variables (e.g., quality and distribution) positively influence consumer choice and the purchase frequency of organic meat. The finding emphasizes the effectiveness of green marketing [84] and the important role of retailers in enhancing organic meat purchase and consumption. Furthermore, the significant findings regarding green marketing practices and perceived barriers highlight the need for investigating contextual and environmental factors in explaining consumer purchase of organic food.

This study has also extended the findings of prior studies by comprehensively examining various determinants of attitudes towards organic food purchase. The findings echo the extant literature that suggest that environmental concern [13,50], health consciousness [11,56], food safety concern [43] and organic food knowledge [44,58] significantly strengthen attitudes towards purchasing organic food. Notably, among the determinants, organic food knowledge has the weakest impact on attitudes. This finding may be due to the low level of knowledge about organic food demonstrated by the respondents in this study (Mean = 3.87). As such, the respondents did not comprehensively understand the unique benefits and characteristics of organic food, which might have adversely affected their attitudes towards buying the product. It is also interesting to note that environmental concern has the strongest influence on attitudes. Several authors suggest that consumers in poorer countries, like developing and emerging markets (e.g., Vietnam), are less likely to take environmental quality into account when making a purchase decision [38,60]. This finding can be partly explained by the fact that the respondents were urban consumers, the majority of who were middle and high income earners. These consumers are likely to care more about the environment. Hence, generalizing this finding to Vietnam as a whole should be undertaken with caution. The finding may also suggest that Vietnamese consumers have increasingly expressed environmental concerns through their purchase decisions associated with eco-friendly products, such as organic meat.

This study’s findings suggest several important implications for marketers, policy makers, organic food associations and socio-environmental organizations that seek to develop intervention strategies aimed at fostering organic food purchases among Vietnamese consumers. Firstly, the demand for organic meat might be encouraged by making such products less costly in terms of value for money. In this regard, organic food producers should make every effort to increase their efficiencies, which would result in lower prices, whilst distributors should consider discounting the price of organic meat whenever possible. Secondly, food stores need to improve the effectiveness of their green marketing practices. It is vital that organic meat becomes more widely available and that in-store communications utilizing fliers, signages and staff are better implemented. Policy makers should facilitate the development and implementation of the national organic labelling program. Thirdly, these initiatives should be supported by intervention strategies aimed at enhancing consumer attitude towards purchasing organic food. This might be desirable in the long run because of two main reasons. The first reason is that although the relationship between attitude and purchase behavior is insignificant, attitude still has a (weak) positive effect on purchase behavior. The second reason is that, improved and strengthened attitudes could be gradually translated into actual consumer purchase of organic meat. Hence, education and communication programs should be jointly developed by the stakeholders to increase consumers’ knowledge about organic meat as well as their awareness about the safety, environmental and health benefits of organic meat.

However, this study is not without its limitations, which can be classified into four broad areas. First, although the sample size is relatively large, the generalizability of the current study is still limited owing to the fact that the sample consisted of urban consumers in only one city, i.e., Hanoi. Second, whilst beyond the scope of this study, interrelationships may exist between the model’s independent variables (e.g., between environmental concern and price barriers). Third, hindrances to organic meat purchase were represented only by price barriers. Finally, this study obtained data during one particular time period, an issue which underestimates the dynamics of variables such as environmental concern, attitude and behavior.

## 6. Conclusions and Future Research

This study is among the first of its kinds that comprehensively investigated factors influencing consumer attitude and purchase of organic meat in the context of Vietnam. The findings highlighted the attitude-behavior gap associated with pro-environmental behavior and organic food consumption. This gap can be partially explained by the negative impact of price barriers on consumer purchase behavior. The findings also concluded that whilst environmental concern has the greatest influence on attitudes towards purchasing organic food, knowledge about organic food has the weakest impact on such attitudes. These recent findings extended current knowledge about organic food purchase in emerging markets as well as provided governmental organizations, marketers and socio-environmental organizations with valid suggestions on how to foster and enhance consumer attitudes and behavior towards organic food products, including organic meat.

The validated research model in this study combined personal factors (i.e., environmental concern, health consciousness, food safety concern and organic food knowledge) with contextual and environmental factors (i.e., food stores’ green marketing and price barriers) to better explain consumers’ decisions relating to organic food. It therefore can serve as a framework for future research in other emerging market economies. Furthermore, to address the limitation of this study’s sample, future research should obtain data from respondents located in other major cities such as Hai Phong, Da Nang and Ho Chi Minh city. It might be desirable to collect data from consumers in rural areas, which enables a comparative analysis between rural (lower income) and urban (higher income) consumers. Future research could also examine different barriers to organic food purchases, such as habits, skepticism on organic food labels, lack of knowledge and availability. This will certainly provide a more comprehensive understanding of the influence of situational factors and the attitude-behavior gap. In addition, it would be beneficial to investigate the interrelationships between the determinants of consumer attitudes about organic food to further address the motivational complexity of organic food consumption. Finally, future research could investigate changes in organic food attitudes and behavior over time by conducting a longitudinal study.

## Figures and Tables

**Table 1:** Demographic profile of the respondents.

Demographic Characteristic	*Frequency*	%
*Gender*		
Female	319	52.4
Male	290	47.6
*Age*		
18–29	180	29.6
30–39	151	24.8
40–49	139	22.8
50–59	87	14.3
60 and above	52	8.5
*Marital status*		
Single/never married	172	28.3
Currently married	376	61.7
Widowed	34	5.6
Divorced/separated	27	4.4
*Educational level*		
High school or lesser	35	5.8
Professional degree	77	12.6
College degree	120	19.7
University undergraduate	276	45.3
Postgraduate	101	16.6
*Household monthly income*		
Under VND 10,000,000	181	29.7
VND 10,000,001–20,000,000	237	38.9
VND 20,000,001–30,000,000	83	13.6
VND 30,000,001–40,000,000	46	7.6
VND 40,000,001–50,000,000	41	6.7
Over VND 50,000,000	21	3.5

Note: US$ 1 = Vietnamese Dong (VND) 23,390 at the time of the survey.

**Table 2:** Items, reliability and convergent validity.

Variables and Items	FLs	α	CR	AVE
*Environmental concern*		0.848	0.850	0.586
The balance of nature is very delicate and can be easily upset	0.769			
Human beings are severely abusing the environment	0.797			
Humans must maintain the balance with nature in order to survive	0.800			
Human interferences with nature often produce disastrous consequences	0.692			
*Food safety concern*		0.802	0.805	0.580
Nowadays most foods contain residues from chemical sprays and fertilizers	0.754			
I am very concerned about the number of antibiotics, veterinary residues and preservatives in meat	0.819			
The quality and safety of meat nowadays concerns me	0.708			
*Health consciousness/concern*		0.794	0.795	0.564
I choose meat carefully to ensure good health	0.775			
I think of myself as a health-conscious consumer	0.779			
I think often about health issues	0.697			
*Organic food knowledge*		0.822	0.822	0.536
In comparison with an average person I know a lot about organic meat	0.782			
I know a lot about how to judge the quality of organic meat	0.720			
I know a lot about the environmental and health benefits of organic meat	0.722			
People who know me, consider me as an expert in the field of organic meat	0.701			
*Attitude towards buying organic food*		0.828	0.829	0.548
Buying organic meat instead of conventional meat is beneficial	0.787			
Buying organic meat instead of conventional meat is a wise choice	0.716			
Buying organic meat instead of conventional meat make me feel good	0.743			
Buying organic meat instead of conventional meat make me feel pleased	0.713			
*Green marketing practices*		0.845	0.845	0.577
This store distributes published fliers of organic meat	0.769			
This store deals with organic meat with certified labels	0.731			
There is environmentally-friendly shopping space in this store	0.789			
This store increases sales of brands of organic meat	0.749			
*Perceived monetary barriers*		0.777	0.778	0.538
Organic meat is still too expensive	0.727			
The price of organic meat is a barrier to purchase it	0.729			
People should buy organic meat, even though they are more expensive than conventional meat (reverse in coding)	0.745			

Note: FLs: factor loadings; α: Cronbach’s alpha; CR: composite reliability; AVE: average variance extracted.

**Table 3:** Descriptive statistics and discriminant validity.

Constructs	Mean	SD	ENV	SAF	HEA	KNO	ATT	GMA	BAR
Environmental concern—ENV	4.665	1.226	0.766						
Food safety concern—SAF	4.859	1.090	0.321	0.762					
Health consciousness—HEA	4.856	1.198	0.295	0.212	0.751				
Organic food knowledge—KNO	3.870	1.084	0.295	0.256	0.290	0.732			
Attitude—ATT	4.644	1.207	0.420	0.368	0.354	0.274	0.740		
Green marketing practices—GMA	4.586	1.147	0.342	0.278	0.224	0.334	0.448	0.760	
Price barriers—BAR	3.239	1.069	−0.408	−0.418	−0.235	−0.419	−0.491	−0.393	0.734

Note: Diagonal value indicates the square root of AVE of construct; SD: standard deviation.

**Table 4:** SEM (structural equation modelling) results and hypotheses testing.

Hypotheses			*β*	S.E.	*t*-Value	*p*-Value	Findings
H1: Environmental concern	→	Attitude	0.273	0.053	5.480	***	Supported
H2: Food safety concern	→	Attitude	0.232	0.057	4.726	***	Supported
H3: Health consciousness	→	Attitude	0.205	0.052	4.151	***	Supported
H4: Knowledge	→	Attitude	0.097	0.053	2.007	0.045	Supported
H5: Attitude	→	Purchase behavior	0.049	0.047	1.189	0.234	Not supported
H6: Green marketing	→	Purchase behavior	0.430	0.058	9.416	***	Supported
H7: Price barriers	→	Purchase behavior	−0.194	0.069	−4.156	***	Supported

Note: *** *p* < 0.001; S.E.: standard error.
